# 1899. Core Outcome Domains for *Mycobacterium avium* complex Pulmonary Disease Clinical Trials: *Mycobacterium avium complex* Core Outcomes Research (MACCOR)

**DOI:** 10.1093/ofid/ofad500.1727

**Published:** 2023-11-27

**Authors:** Cara D Varley, Ryan Stadnik, Clifton Bingham, Alexandra L Quittner, David Lewinsohn, Kevin L Winthrop

**Affiliations:** Oregon Health and Science University, Portland, Oregon; OHSU, Portland, Oregon; Johns Hopkin s University, Baltimore, Maryland; Joe DiMaggio Children's Hospital, Miami, Florida; Oregon Health & Science University, Portland, Oregon; OHSU-PSU School of Public Health, Portland, Oregon

## Abstract

**Background:**

*Mycobacterium avium complex* pulmonary disease (MAC-PD) is a chronic, inflammatory disease with systemic manifestations affecting multiple aspects of patients’ lives. It can require lifelong management, as recurrence is common, however current microbiologic outcomes are often difficult to obtain and insufficient to reflect the impact on patients. We aim to identify the core outcome domains essential to evaluate in future clinical research studies of outcomes in MAC-PD, using an international consensus process, and we describe the Round 1 preliminary results.

**Methods:**

We invited relevant stakeholders in MAC-PD research including patients, caregivers/family members, clinicians, researchers, nonprofit and patient education organizations, and federal research funding organizations to participate in the Delphi consensus process. We used respondent driven sampling to expand our cohort. Participants were asked to rate preliminary domain importance, without regard to availability, feasibility, or validity of measurement instruments, using the Grading of Recommendations Assessment, Development and Evaluation scale (score 1-9). We administered surveys via email through DelphiManager software. We used SAS version 9.4 to describe our participants and the Round 1 core outcome domain scores.

**Results:**

We had a total of 48 participants, including 30 (62.5%) clinicians, 10 (20.8%) researchers, 6 (12.5%) patients, and 2 (4.2%) from organizations that fund MAC-PD research. A majority were women (27, 56.3%), ages 60-69 (16, 33.3%) who identified as White (39, 81.3%) and were from North America (45, 93.8%). Most had 10 years or more experience (33, 68.8%) in their MAC-PD stakeholder role. Symptoms, Microbiology, Treatment Side effects, Chest imaging, Physical function, Treatment burden, and Vitality/Energy domains were rated as *Critical* (score >7) by more than 70% and had fewer than 15% identifying the domain as *Not Important* (score <3).

**Figure:**
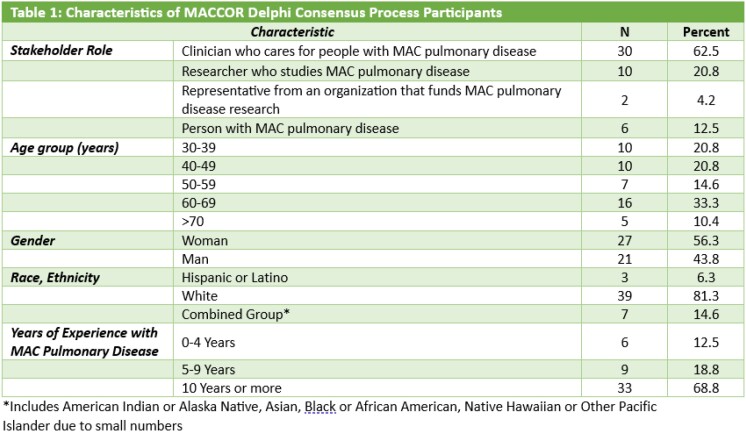

**Figure:**
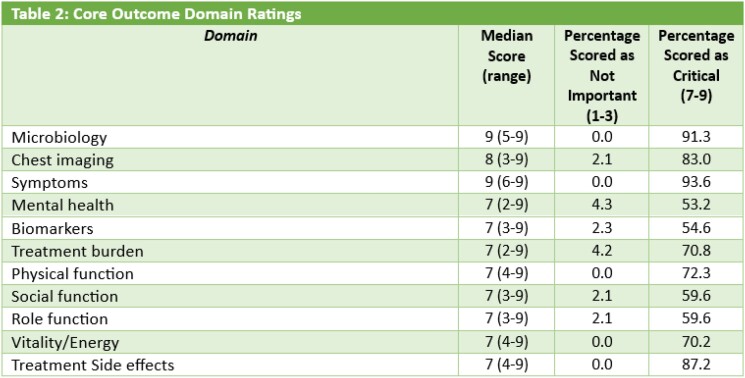

**Conclusion:**

We report the initial evaluation of core outcome domains for MAC-PD through an international consensus process. Identification and dissemination of these domains will help guide research priorities on future outcome measures.

**Figure:**
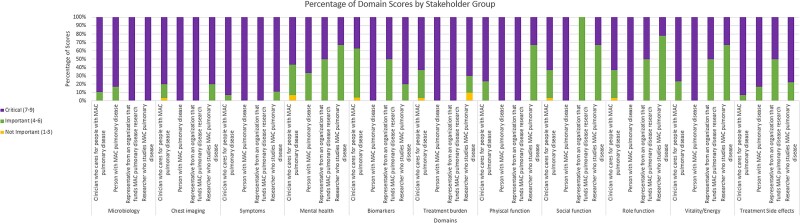

**Disclosures:**

**Alexandra L. Quittner, PhD**, AN2: Advisor/Consultant|Insmed Pharmaceutical Corp: Advisor/Consultant **Kevin L. Winthrop, MD, MPH**, AN2: Advisor/Consultant|AN2: Grant/Research Support|Insmed: Advisor/Consultant|Insmed: Grant/Research Support|Insmed: This study was funded by Insmed Inc.|Paratek: Advisor/Consultant|Paratek: Grant/Research Support|Red Hill Biopharma: Advisor/Consultant|Red Hill Biopharma: Board Member|Red Hill Biopharma: Grant/Research Support|Renovion: Advisor/Consultant|Renovion: Grant/Research Support|Spero: Advisor/Consultant|Spero: Grant/Research Support

